# In vitro assessment of hepatoprotective agents against damage induced by acetaminophen and CCl_4_

**DOI:** 10.1186/s12906-016-1506-1

**Published:** 2017-01-13

**Authors:** Liliana Torres González, Noemí Waksman Minsky, Linda Elsa Muñoz Espinosa, Ricardo Salazar Aranda, Jonathan Pérez Meseguer, Paula Cordero Pérez

**Affiliations:** 1 Liver Unit, Gastroenterology Service, Department of Internal Medicine, University Hospital “Dr. José E. González”, Av. Gonzalitos #235 Col. Mitras Centro C.P., 64460 Monterrey, Nuevo León Mexico; 2 Department of Analytical Chemistry, School of Medicine, Universidad Autónoma de Nuevo León, Av. Madero y Aguirre Pequeño S/N, Col. Mitras Centro, C. P., 64460 Monterrey, N. L. Mexico

**Keywords:** Hepatoprotective, HepG2 cell line, Acetaminophen, Carbon tetrachloride, Silybinin, Silyphos, Silymarin

## Abstract

**Background:**

In vitro bioassays are important in the evaluation of plants with possible hepatoprotective effects. The aims of this study were to evaluate the pretreatment of HepG2 cells with hepatoprotective agents against the damage induced by carbon tetrachloride (CCl_4_) and paracetamol (APAP).

**Methods:**

Antioxidative activity was measured using an assay to measure 2,2-diphenyl-1-picrylhydrazyl (DPPH) free radical scavenging. The in vitro hepatotoxicity of CCl_4_ and APAP, and the cytotoxic and hepatoprotective properties of silymarin (SLM), silybinin (SLB), and silyphos (SLP) were evaluated by measuring cell viability; activities of aspartate aminotransferase (AST), alanine aminotransferase (ALT), and lactate dehydrogenase (LDH); total antioxidant capacity (TAOxC); and reduced glutathione (GSH), superoxide dismutase (SOD), and lipid peroxidation (malondialdehyde (MDA) levels).

**Results:**

Only SLB and SLM showed strong antioxidative activity in the DPPH assay (39.71 ± 0.85 μg/mL and 14.14 ± 0.65 μg/mL, respectively). CCl_4_ induced time- and concentration-dependent changes. CCl_4_ had significant effects on cell viability, enzyme activities, lipid peroxidation, TAOxC, and SOD and GSH levels. These differences remained significant up to an exposure time of 3 h. APAP induced a variety of dose- and time-dependent responses up to 72 h of exposure. SLM, SLB, and SLP were not cytotoxic. Only SLB at a concentration of 100 μg/mL or 150 μg/mL significantly decreased the enzyme activities and MDA level, and prevented depletion of total antioxidants compared with CCl_4_.

**Conclusions:**

CCl_4_ was more consistent than APAP in inducing cell injury. Only SLB provided hepatoprotection. AST, LDH, and MDA levels were good markers of liver damage.

## Background

Medicinal plants with hepatoprotective activity contain a large number of bioactive molecules. The identification of these molecules contained in a biomass complex requires careful selection and execution of appropriate bioassays during the various stages of the research process [1]. In vitro bioassays are important in the evaluation of plants with possible hepatoprotective effects.

Human hepatoma cell lines have been proposed as an alternative to human hepatocytes for in vitro models of normal liver cells. The potential advantages of hepatoma cells are that, as an immortalized cell line, they are readily available in large quantities, they are easy to maintain because they can be cryopreserved, and their drug-metabolizing enzyme activities do not decrease in cultivation, as happens in primary cultures of human hepatocytes [2]. However, an obvious disadvantage is that the mechanisms underlying drug metabolism and toxicity may be abnormal in transformed cells. Despite these issues, the HepG2 hepatoma cell line is used widely in studies of liver function, metabolism, and drug toxicity [3, 4]. HepG2 cells also possess many of the biochemical and morphological characteristics of normal hepatocytes [5]. Because they retain many characteristics of normal liver cells, these cells are used in studies to determine whether medicinal plants have hepatoprotective activities [6, 7].

One hepatoprotective agent used widely in the treatment of various liver disorders, such as hepatitis or fatty infiltration caused by alcohol or toxins, is the standardized extract of *Silybum marianum*, known as milk thistle or silymarin (SLM) [8–11]. It is a complex mixture of the flavonolignans silybinin (SLB), silychristin, silydianin, and isosilybin. SLB, a polyphenolic molecule, is the major component of SLM and is responsible for its pharmacological activity [12, 13]. SLM is poorly absorbed, although the bioavailability of SLB is higher than that of phosphatidylcholine (silyphos (SLP)) [14, 15].

The major inducers of hepatic damage used when evaluating hepatoprotective activity are paracetamol (acetaminophen, APAP) and carbon tetrachloride (CCl_4_). However, there are few reports on their use and in vitro characteristics [6, 7, 16]. The mechanisms responsible for the in vivo liver toxicity of both compounds are complex and involve several cell types [17, 18]. CCl_4_ undergoes metabolic activation in a cytochrome P-450-dependent step to produce free radicals, which can initiate lipid peroxidation. The toxicity induced by CCl_4_ in vivo and in cultured hepatocytes involves stimulation of lipid peroxidation, which is detected as an increase in malondialdehyde (MDA) formation [19]. APAP is metabolized mainly in the liver to excretable glucuronide and sulfate conjugates. However, the hepatotoxicity of APAP has been attributed to the formation of toxic metabolites, which occurs when APAP is activated by hepatic cytochrome P-450 [20] to a highly reactive metabolite N-acetyl-P-benzoquinoneimine (NAPQI) [21]. NAPQI is initially detoxified by conjugation with reduced glutathione (GSH) to form mercapturic acid. However, when its rate of formation exceeds the rate of detoxification by GSH, NAPQI oxidizes tissue macromolecules such as lipids and —SH group proteins, and calcium homeostasis is altered by depletion of GSH [22].

The aims of this study were to evaluate the hepatoprotective activities of SLM, SLB, and SLP against liver damage induced by APAP and CCl_4_ in the HepG2 cell line.

## Methods

### General

SLB, SLM, 2,2-diphenyl-1-picrylhydrazyl free radical (DPPH), 3-(4,5-dimethylthiazol-2-yl)-2,5-diphenyl-2H-tetrazolium bromide (MTT), and CCl_4_ (99.9%) were purchased from Sigma-Aldrich Chemical Co. (St Louis, MO, USA). SLP was purchased from Medix, S.A. de C.V. (México City, D.F. México), and dimethyl sulfoxide (DMSO) was purchased from ACS Research Organics (Cleveland, OH, USA). Total antioxidant capacity (TAOxC), GSH, superoxide dismutase (SOD), and thiobarbituric acid reactive substances were purchased from Kit OXItek (Buffalo, NY, USA). Dulbecco’s modified Eagle’s medium advanced (DMEMA) with and without phenol red, fetal bovine serum, trypsin 0.25% (1×), penicillin G (100 IU/mL), streptomycin (100 μg/mL), and phosphate-buffered saline (PBS) were purchased from Gibco Invitrogen (Carlsbad, CA, United States). Aspartate aminotransferase (AST), alanine aminotransferase (ALT), and lactate dehydrogenase (LDH) activities were measured using an ILab 300 Plus chemistry analyzer (Instrumentation Laboratory, Bedford, MA, USA).

### Measurement of free radical reduction using the DPPH assay

Antioxidant activity was measured as described previously by Salazar et al. [23]. Briefly, the hepatoprotective agents were dissolved in ethanol to obtain stock solutions (1000 μg/mL), from which serial dilutions were made. Diluted solutions (0.5 mL of each) were mixed with 0.5 mL of 125 μM DPPH and allowed to react for 30 min. Ultraviolet absorbance was recorded at 517 nm (Multiskan EX; Thermo/LabSystems, Vantaa, Finland). The experiment was performed in triplicate and the average absorption was recorded for each concentration. The same procedure was followed for the quercetina (positive control).

### Cell culture

HepG2 human liver hepatoma cells were obtained from the Laboratory of Liver, Pancreas and Motility, Department of Experimental Medicine, Faculty of Medicine, Universidad Nacional Autónoma de México, México City, DF, México. Cells were grown in standard conditions: supplemented DMEMA at 37 °C in a humidified 5% carbon dioxide atmosphere. When the cells reached 80–90% confluence, they were trypsinized and plated at 30,000 cells per well in a 96-well microplate, 1 × 10^6^ cells per well in six plates, or 5 × 10^7^ cells per well in a single dish, depending on the determination. The cells were used after attachment.

### CCl_4_-induced toxicity in HepG2 cells

HepG2 cells were incubated in medium or treated with the toxic agent (20 mM, 30 mM, or 40 mM CCl_4_ in 0.05% DMSO) for 1, 1.5, 2, or 3 h. The evaluation assays were performed using standard methods as described in the “Evaluation assays” section below.

### APAP-induced toxicity in HepG2 cells

HepG2 cells were treated with the toxic agent (2 mM, 4 mM, or 8 mM APAP) or incubated with medium only for 12, 24, 48, or 72 h. The evaluation assays were performed using standard methods as described in the “Evaluation assays” section below.

### Effects of SLM, SLB, and SLP on HepG2 cells

The cytotoxic effects of SLM, SLB, and SLP were measured in HepG2 cells exposed for 12 h to compounds at 10, 100, or 150 μg/mL in supplemented DMEMA. HepG2 cells in medium only were used as a negative control. The evaluation assays were performed using standard methods as described in the “Evaluation assays” section below.

### In vitro assay to identify hepatoprotective effects

The hepatoprotective effects of SLM, SLB, and SLP on HepG2 cells were measured as follows. Normal control cells were incubated with DMEMA in DMSO (0.05% v/v) for 12 h. For toxic treatment, cells were incubated with DMEMA in DMSO (0.05% v/v) for 12 h and then treated with DMEMA with 40 mM CCl_4_ for 1.5 h. For SLM treatment, cells were incubated with DMEMA with SLM at 10, 100, or 150 μg/mL for 12 h and then treated with 40 mM CCl_4_ for 1.5 h. For SLB treatment, cells were incubated with DMEMA with SLB at 10, 100, or 150 μg/mL for 12 h and then treated with 40 mM CCl_4_ for 1.5 h. For SLP treatment, cells were incubated with DMEMA with SLP at 10, 100, or 150 μg/mL for 12 h and then treated with 40 mM CCl_4_ for 1.5 h. The evaluation assays were performed using standard methods as described in the “Evaluation assays” section below.

### Evaluation assays

Each assay was performed in triplicate and the experiments were repeated three times.

### Cell viability assay

Cell viability was assessed using the MTT reduction assay with slight modifications [24]. This colorimetric assay involves the conversion of MTT to a purple formazan derivative by mitochondrial succinate dehydrogenase, which is present only in viable cells. The cells were treated with SLM, SLB, SLP, and/or the toxic agent. The medium was then removed and the cells were then incubated with MTT (0.5 mg/mL) for 2 h, after which the formazan crystals were dissolved with 200 μL/well of DMSO. Absorbance was measured at 570 nm (Multiskan EX; Thermo/LabSystems, Vantaa, Finland). Viability was defined as the ratio of the absorbance of treated cells to that of untreated control cells and is expressed as a percentage.

### Measurement of AST, ALT, and LDH activities

AST, ALT, and LDH activities were measured using an ILab 300 Plus system and Instrumentation Laboratory assay kits. HepG2 cells were treated with SLM, SLB, SLP, and/or the toxic agent. The supernatant was removed from the wells, and the enzyme activities were measured immediately. The results are expressed as IU/L.

### Measurement of TAOxC

TAOxC was measured in lysed HepG2 cells using an Antioxidant Assay kit from Cayman Chemical Company (Ann Arbor, MI, USA). The kit is based on the ability of antioxidants in the sample to inhibit the oxidation of 2,2’-azino-bis-3-ethylbenzothiazoline (ABTS) to ABTS^+^ by metmyoglobin. HepG2 cells were treated with SLM, SLB, SLP, and/or the toxic agent. After treatment, the adherent cells were scraped off and suspended in 5 mM potassium phosphate, pH 7.4, containing 0.9% sodium chloride and 0.1% glucose, sonicated, and placed on ice. The supernatant of the lysed cells was used to measure TAOxC. Absorbance in the well was measured after 5 min at a wavelength of 405 nm on a microplate reader (Multiskan EX; Thermo/LabSystems, Vantaa, Finland). The results are expressed as millimoles of antioxidant.

### Measurement of GSH level

GSH level was quantified using a Glutathione Assay kit from Cayman Chemical Company. The assay kit is based on the enzymatic 5,5’-dithiobis-2-(nitrobenzoic acid) (DTNB) disulfide dimer-oxidized GSH reductase recycling method. After treatment, the medium was removed from the wells, and the adherent cells were scraped off and suspended in 0.5 mL of 50 mM phosphate, pH 6.5, containing 1 mM ethylenediaminetetraacetic acid, sonicated, and placed on ice. The supernatant of lysed cells was used to measure GSH level. Absorbance of the yellow product in the well was measured at a wavelength of 405 nm on a microplate reader at 5 min intervals for 30 min. The total GSH activity was measured using the kinetic method from a standard curve of GSH. The results are expressed as micromoles of GSH per liter.

### Measurement of SOD activity

SOD activity was measured using a Superoxide Dismutase Assay kit from Cayman Chemical Company, which uses a colorimetric assay to measure the concentration of formazan crystals. This assay uses a tetrazolium salt for the detection of superoxide radicals generated by xanthine oxidase and hypoxanthine. After treatment, the medium was removed from the wells, the adherent cells were scraped off and suspended in 20 mM HEPES buffer, pH 7.2, containing 1 mM EGTA, 210 mM mannitol, and 70 mM sucrose), sonicated, and placed on ice. To measure SOD activity, the diluted radical detector and the supernatant of lysed cells or standard were added to each well of a 96-well plate, and xanthine oxidase was added. Absorbance in the well was measured at a wavelength of 460 nm after 20 min on a microplate reader. The results are expressed as IU/mL.

### Measurement of lipid peroxidation

The concentration of MDA, the end product of lipid peroxidation, was measured using a thiobarbituric acid reactive substance (TBARS) Assay kit from Cayman Chemical Company. After treatment, the medium was removed from the wells, adherent cells were scraped off, suspended in cold PBS, sonicated, and placed on ice. The supernatant from lysed cells or standard, sodium dodecyl sulfate, and the color reagent were added to each vial. The vial was heated at 100 °C for 1 h and then immediately cooled in an ice bath and centrifuged. The content of each vial was transferred to a well in a microplate. The absorbance of the product was measured at a wavelength of 540 nm on a microplate reader. The extent of lipid peroxidation was quantified by estimating the MDA concentration. The results are expressed as micromoles of MDA equivalents formed per liter.

### Statistical analysis

The half-maximal inhibitory concentration (IC_50_) values were calculated by regression analysis. The results are expressed as mean ± standard deviation (SD). The data were analyzed using one-way analysis of variance (ANOVA) followed by Dunnett’s multiple-comparison test using Prism software (v. 6.0; GraphPad, San Diego, CA, USA). Differences between means were considered significant at *P* <0.05.

## Results

### DPPH radical-scavenging activity

The DPPH radical-scavenging activity of SLM, SLB, and SLP was evaluated. The IC_50_ values were 39.71 ± 0.85 μg/mL, 14.14 ± 0.65 μg/mL, and 169.53 ± 2.19 μg/mL, respectively. Quercetin (used as a positive reference) scavenged DPPH radicals completely, and its IC_50_ value was 2 μg/mL.

### CCl_4_-induced toxicity in HepG2 cells

**Fig. 1:**
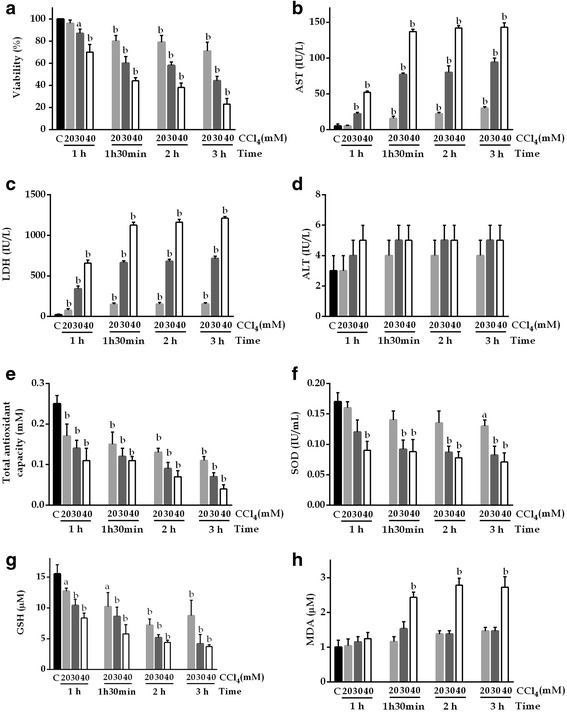
Time-dependent changes in HepG2 cells. **a** Cell viability, **b** AST, **c** LDH, **d** ALT, **e** TAOxC, **f** SOD, **g** GSH, and **h** MDA levels after exposure to 20, 30, or 40 mM CCl_4_. Control: DMSO (0.05% v/v) in supplemented DMEMA; CCl_4_ 20 mM: 1.92 μL of CCl_4_/DMSO (0.05% v/v) in supplemented DMEMA; CCl_4_ 30 mM: 2.88 μL of CCl_4_/DMSO (0.05% v/v) in supplemented DMEMA; CCl_4_ 40 mM: 3.84 μL of CCl_4_/DMSO (0.05% v/v) in supplemented DMEMA. Values are the mean ± SD of three independent experiments performed in triplicate. ^a^ *P* <0.05 vs C; ^b^ *P* <0.01 vs C

The toxic effects of CCl_4_ were time and concentration dependent (Fig. 1). Compared with the vehicle control, there were significant differences in cell viability; AST, ALT, and LDH activities; lipid peroxidation; TAOxC; and SOD and GSH levels. These differences remained significant up to an exposure time of 3 h (*P* <0.01).

### APAP-induced toxicity in HepG2 cells

**Fig. 2:**
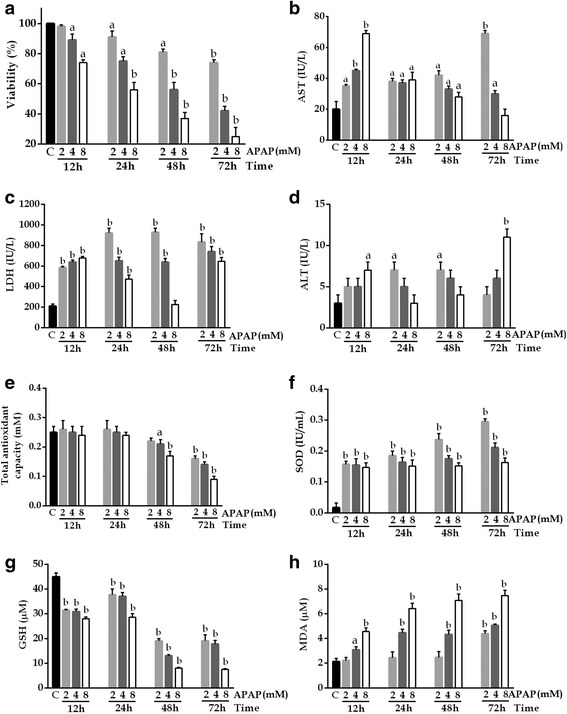
Changes after exposure to 2, 4, or 8 mM APAP in HepG2 cells. **a** Cell viability, **b** AST, **c** LDH, **d** ALT, **e** TAOxC, **f** SOD, **g** GSH, and **h** MDA levels. Control: supplemented DMEMA; APAP 2 mM: 2 μL in supplemented DMEMA; APAP 4 mM: 4 μL in supplemented DMEMA; APAP 8 mM: 8 μL in supplemented DMEMA. Values are the mean ± SD of three independent experiments performed in triplicate. ^a^ *P* <0.05 vs C; ^b^ *P* <.01 vs C

The toxic effects of APAP are shown in Fig. 2. The release of AST, ALT, and LDH increased during the first 12 h in HepG2 cells exposed to APAP, after which it declined; at 72 h, the effect was not related to time or concentration. TAOxC and SOD and GSH concentrations, and cell viability decreased and MDA concentration increased in a dose- and time-dependent manner. These changes remained significant up to an exposure time of 72 h (*P* <0.01).

### Effects of SLM, SLB, and SLP in HepG2 cells

**Fig. 3:**
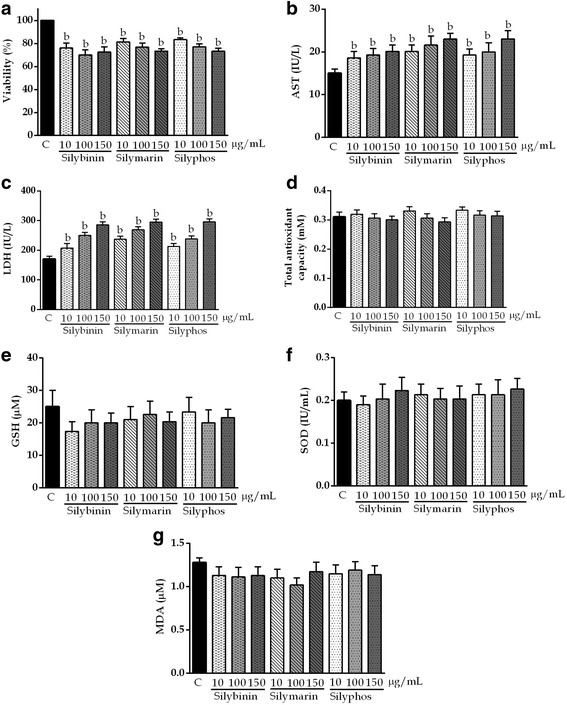
Effects of silybinin, silymarin, and silyphos at concentrations of 10, 100, and 150 μg/mL for 12 h in HepG2 cells. The values are the mean ± SD. ^a^ *P* <0.05 vs C; ^b^ *P* <0.01 vs C

The cytotoxic effects of SLM, SLB, and SLP on HepG2 cells exposed for 12 h are shown in Fig. 3. The compounds were considered to be toxic if there was a >60% decrease in cell viability compared with untreated cells, an AST level >50 IU/L, ALT level >30 IU/L, TAOxC >2 mM, or an MDA, SOD, or GSH concentration greater than that of the control (criteria established previously) [25]. According to these definitions, the compounds were not considered cytotoxic and were used to evaluate hepatoprotective activity.

### In vitro hepatoprotective effects

**Fig. 4:**
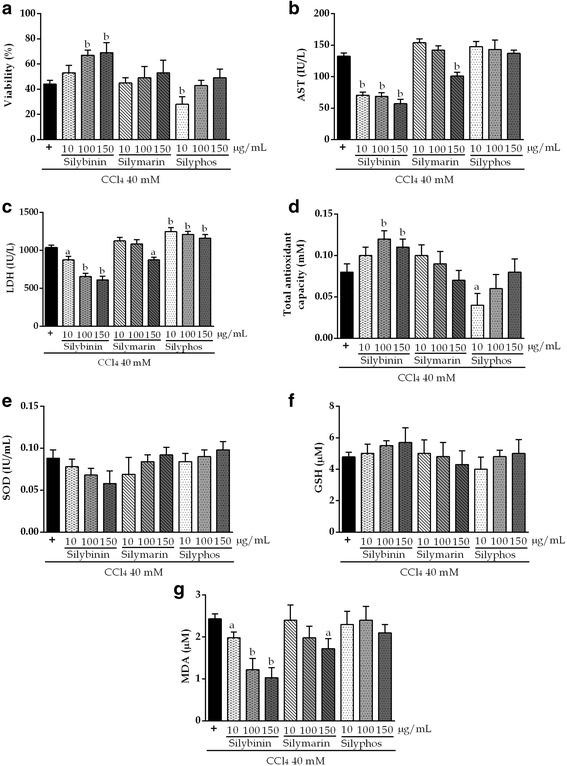
Hepatoprotective effects of silybinin, silymarin, and silyphos against damage induced by 40 mM CCl_4_ for 1.5 h in HepG2 cells. The values are the mean ± SD. ^a^ *P* <0.05 vs CCl_4_; ^b^ *P* <0.01 vs CCl_4_

The hepatoprotective effects of SLM, SLB, and SLP on HepG2 cells are shown in Fig. 4. HepG2 cells were pretreated with a hepatoprotective agent and subsequently exposed to CCl_4_ to induce damage. Only SLB at a concentration of 100 μg/mL or 150 μg/mL significantly decreased the levels of AST, LDH, and MDA, and prevented depletion of TAOxC compared with CCl_4_ (*P* <0.01). Pretreatment with SLM at 10 or 100 μg/mL and SLP at any concentration did not prevent the reduction in TAOxC compared with CCl_4_. Pretreatment with SLM only at 150 μg/mL reduced the enzyme levels compared with CCl_4_ (*P* <0.01).

## Discussion

In this study, we used the HepG2 cell line to evaluate the hepatoprotective activity of SLM, SLB, and SLP against liver damage induced by APAP and CCl_4_ in an attempt to establish a simple strategy for monitoring the hepatoprotective activities of plant extracts without high-end and excessive testing.

Cells exposed to these toxic agents lose cell viability, release liver enzymes into the culture medium, do not metabolize the tetrazolium salt, and exhibit significantly changed TAOxC and levels of MDA, SOD, and GSH [16, 26]. The in vitro liver damage caused by CCl_4_ has been hypothesized to be caused by two different mechanisms, depending on the concentration used and the exposure time: a direct solvent effect of the molecule itself or an indirect effect through the generation of free radicals and subsequent lipid peroxidation [27].

Berger et al. studied the induction of cell membrane damage in isolated rat hepatocytes during the first 10–30 min of exposure to 20% CCl_4_ in ethanol by quantifying the MDA level as a marker of lipid peroxidation [28]. They postulated that these changes were caused by the direct action of the solvent, which affected the cell membrane, and that such changes are not preventable by antioxidant treatment within the initial 30 min of exposure. In the current study, CCl_4_ did not cause toxic effects or increase MDA level within 30 min, but effects were seen at 60 min; this observation seems to exclude any direct solvent effect. Holden et al. reported that the toxic effect of CCl_4_ at a concentration of 0.18% in HepG2 cells, as measured by release of LDH into the culture medium, increased significantly beginning at 1.5 h and LDH increased the release by up to 50% [26]. In our study, compared with control cells, HepG2 cells exposed to CCl_4_ exhibited a similar LDH response at 1.5 h.

One study found significant participation of lipid peroxidation in the toxic effects via formation of free radicals and loss of viability in these cells after exposure to 0.5% CCl_4_ for 2 h [16]. We found that, compared with control cells, HepG2 cells exposed to CCl_4_ showed significantly reduced viability, reduced TAOxC and GSH and SOD levels, and increased AST, LDH, and MDA activities at 1.5 h. ALT activity did not change significantly, although this lack of change may relate to the timing of exposure to the toxic agent because previous studies have shown time-dependent increments in ALT activity [6, 7].

Oxidative stress also plays a major role in APAP toxicity. Oxidative stress occurs when the generation of reactive oxygen species overwhelms the ability to detoxify the reactive intermediates or exceeds the capacity to repair the resulting damage [29]. It has been reported that high APAP levels cause injury and reduce viability by up to 80% at 24 h [30]. In our study, HepG2 cells exposed to APAP showed similar results, and cell death was greatest after the longest exposure time (72 h). In other studies, HepG2 cells were exposed to serial concentrations of APAP for 24 and 72 h, and LDH release increased with a dose of 10 mM [31]. By contrast, in our study, increases in AST, ALT, and LDH release were evident only during the first 12 h. However, the decreases in the TAOxC and SOD and GSH levels, and the increase in MDA level were time dependent throughout the observation period. HepG2 cells are killed by APAP [32, 33], but the mechanism causing death differs between HepG2 cells and cells that form a reactive metabolite, which causes apoptosis. For this reason, GSH depletion does not occur in HepG2 cells at 12 h [34], and our finding that GSH started to decrease only after 12 h is consistent with this observation. Because of the variability of the results obtained with APAP, the best inducer of cell injury in our study was CCl_4_ after 1.5 h incubation and at a concentration of 40 mM. Experimental results using various mediators of oxidative stress confirm the involvement of free radicals acting through lipid peroxidation, as reported for this toxic agent [6, 7, 16, 26, 28].

The hepatoprotective effects of *S. marianum* are due mainly to its antioxidant content. The DPPH assay is based on the reduction of the stable DPPH radical to a yellow diphenyl picryl hydrazine, which is a common spectrophotometric method for measuring the antioxidant capacity of compounds. Thus, the ability of SLM, SLB, and SLP to quench this radical is a measure of antioxidative activity. In this assay, the antioxidative activity was greater for SLB than for SLM, and for SLM than for SLP. The lower activity of SLP might relate to interactions between the different substances in the compound evaluated (Medix). The antioxidative activities of SLB and SLM that we observed are in agreement with previous research, which has reported strong DPPH free radical-scavenging activity for these compounds [35, 36].

Before evaluating the hepatoprotective activity of the various concentrations of SLM, SLB, and SLP, it was necessary to demonstrate that they are nontoxic. These compounds were not cytotoxic according to the definition of toxicity as a >60% decrease in cell viability compared with untreated cells, AST level <50 IU/L, ALT level <30 IU/L, TAOxC <2 mM or MDA, SOD, or GSH level less than that of the control [25]. This finding is in agreement with those of previous studies [35–37].

We evaluated the hepatoprotective activities of SLM, SLB, and SLP against CCl_4_-induced liver damage at a dose of 40 mM for 1.5 h. In other in vivo models, *S. marianum* was reported to increase GSH level and to decrease MDA level, and SLB was shown to be the major biologically active component of SLM [12, 38, 39]. We found that pretreatment with SLB at the highest doses prevented the biochemical alterations indicative of damage induced by CCl_4_, although SLM did not have significant effects on cell viability at any of the doses studied.

It is important to mention that one way of indirectly assessing the damage to HepG2 cells caused by free radicals is by measuring the activities of intracellular enzymes (e.g., GSH, SOD) and TBARS, and the viability of cultured cells using the MTT assay. These measurements are useful for assessing the in vitro antioxidative actions of the hepatoprotective plant extracts [25, 40–43]. However, it has been proposed that other isolated active compounds in addition to those mentioned above should be included when evaluating the intracellular formation of reactive oxygen species, mitochondrial membrane potential, and changes in cell nuclei morphology in in vitro models. However, the use of all of these compounds may not be practical for routine testing because of the high cost of their inclusion in the monitoring of the hepatoprotective activities of all plant extracts [44–47].

## Conclusion

The findings from this study show that CCl_4_ was a better injury inducer than APAP when used with 1.5 h incubation and at a concentration of 40 mM. SLB at a dose of 150 μg/mL was an adequate positive control for studying hepatoprotection. AST, LDH, and MDA were good markers of liver damage in HepG2 cells.

## Abbreviations

ABTS: 2,2’-azino-bis-3-ethylbenzothiazoline
ALT: Alanine aminotransferase
APAP: Acetaminophen
AST: Aspartate aminotransferase
CCl_4_: Carbon tetrachloride
DMEM-advanced: Dulbecco’s modified Eagle’s medium advanced
DMSO: Dimethyl sulfoxide
DPPH: 2,2-diphenyl-1-picrylhydrazyl free radical
DTNB: 5,5’-dithiobis-2-(nitrobenzoic acid)
GSH: Reduced glutathione
HepG2: Human hepatoma cell line
IC_50_: Half-maximal inhibitory concentration
IU: International unit
LDH: Lactate dehydrogenase
MDA: Malondialdehyde
MTT: 3-(4,5-dimethylthiazol-2-yl)-2,5-diphenyl-2H-tetrazolium bromide
NAPQI: N-acetyl-P-benzoquinoneimine
nm: Nanometer
PBS: Phosphate-buffered saline
SD: Standard deviation
SLB: Silybinin
SLM: Silymarin
SLP: Silyphos
SOD: Superoxide dismutase
TAOxC: Total antioxidant capacity
